# Partial hepatectomy enhances the growth of CC531 rat colorectal cancer cells both in vitro and in vivo

**DOI:** 10.1038/s41598-021-85082-z

**Published:** 2021-03-08

**Authors:** Borja Herrero de la Parte, Mikel González-Arribas, Iñaki Diaz-Sanz, Teodoro Palomares, Ignacio García-Alonso

**Affiliations:** 1 Department of Surgery and Radiology and Physical Medicine, Faculty of Medicine and Nursing, University of the Basque Country (UPV/EHU), Barrio Sarriena s/n, 48940 Leioa, Vizcaya Spain; 2 Biocruces Bizkaia Health Research Institute, Plaza de Cruces s/n, 48903 Barakaldo, Spain

**Keywords:** Cancer, Metastasis, Experimental models of disease, Preclinical research, Cancer

## Abstract

Partial hepatectomy (PHx) is the gold standard for the treatment of colorectal cancer liver metastases. However, after removing a substantial amount of hepatic tissue, growth factors are released to induce liver regeneration, which may promote the proliferation of liver micrometastases or circulating tumour cells still present in the patient. The aim of this study is to assess the effect of PHx on the growth of liver metastases induced by intrasplenic cell inoculation as well as on in vitro proliferation of the same cancer cell line. Liver tumours were induced in 18 WAG/RijHsd male rats, by seeding 250,000 syngeneic colorectal cancer cells (CC531) into the spleen. The left lateral lobe of the liver was mobilized and in half of the animals it was removed to achieve a 40% hepatectomy. Twenty-eight days after tumour induction, the animals were sacrificed and the liver was removed and sliced to assess the relative tumour surface area (RTSA%). CC531 cells were cultured in presence of foetal calf serum, non-hepatectomised (NRS) or hepatectomized rat serum (HRS), and their proliferation rate at 24, 48, and 72 h was measured. RTSA% was significantly higher in animals which had undergone PHx than in the controls (non-hepatectomised) (46.98 ± 8.76% vs. 18.73 ± 5.65%; *p* < 0.05). Analysing each lobe separately, this difference in favour of hepatectomized animals was relevant and statistically significant in the paramedian and caudate lobes. But in the right lobe the difference was scarce and not significant. In vitro, 2.5% HRS achieved stronger proliferative rates than the control cultures (10% FCS) or their equivalent of NRS. In this experimental model, a parallelism has been shown between the effect of PHx on the growth of colorectal cancer cells in the liver and the effect of the serum on those cells in vitro.

## Introduction

Colorectal cancer (CRC) is the major cause of cancer-related morbidity and mortality in developed regions. In the European Union (EU), it is the second leading cause of death, while in developing countries it is the fifth^1,2^. Eurostat reports over 153,000 deaths as a result of CRC in the EU, representing 11% of cancer-associated deaths and 3.1% of all deaths^2,3^.

Metastases are the main cause of CRC-associated morbidity and mortality. The liver is the most common site of metastases derived from CRC (CRCLM), due to the fact that the blood from colon and proximal parts of the rectum drains through the portal system to the liver^4–7^. More than 50% of patients with CRC develop CRCLM in their lifetime^2,6^, and without treatment only approximately 20 to 30% of them survive one year after the diagnosis of the metastatic disease^8^.

Nowadays, partial hepatectomy (PHx) is the only option offering potential cure for patients with CRCLM^9,10^. Regardless of advances in this field, less than 20% of patients are eligible for PHx^11^. If performed with curative intent, PHx can achieve a 5-year survival rate of 23 to 51% and median survival up to 60 months^11,12^. Conversely, in untreated CRCLM patients, median survival is 3–20 months, with a 1-year survival rate of less than 30%, and 5-year survival rate of less than 5%^8^.

Unfortunately, metastasis recurrence following tumour resection is quite a common event^13^, most frequently resulting from the outgrowth of a residual disease, also called residual metastatic disease^14,15^. Some studies suggest that these tumour microimplants or some circulating tumour cells may be in a kind of “dormant state” which is suddenly abandoned to begin rapid growth^16,17^.

During surgical procedures, such as PHx, the disruption and rearrangement of the extracellular tumour matrix may promote detachment and migration of tumour cells within the remaining hepatic mass (RHM) or its incorporation into the systemic circulation, which facilitates intra- or extrahepatic tumour recurrence^18^. In addition, liver resection leads to relevant increments in the levels of growth factors (GF) and cytokines related with liver regeneration, which can induce changes in the microtumour implant environment. Indeed, It has been previously described that liver regeneration plays a significant role in tumour stimulation and metastasis, and that, in particular, the degree of hepatectomy appears to be a key factor for this dismal effect^19^.

In fact, we have already demonstrated that PHx performed after intrasplenic inoculation of rhabdomyosarcoma S4MH cells increases the number and size of liver implants of these tumour cells; in addition, we have shown that serum obtained from hepatectomized rats as well as several GFs enhance the growth of this tumour cell line in vitro^20,21^. Now, we wanted to explore whether it was something specific to this rhabdomyosarcoma cell line, which is known to be a very aggressive tumour, or rather something common to other tumour cell lines. For this reason, we have adapted our model for a syngeneic CRC cell line (CC531), in order to analyse the potential of hepatectomized rat serum (HRS) to enhance CC531 growth in vitro and to check the effect of 40% PHx on the growth of CC531 cancer cell implants in the liver.

## Results

In this experiment, both in vitro and in vivo studies showed that liver resection is a strong stimulus for CC531 cancer cell proliferation.

### Effect of hepatectomized rat serum on in vitro cell proliferation

**Figure 1:**
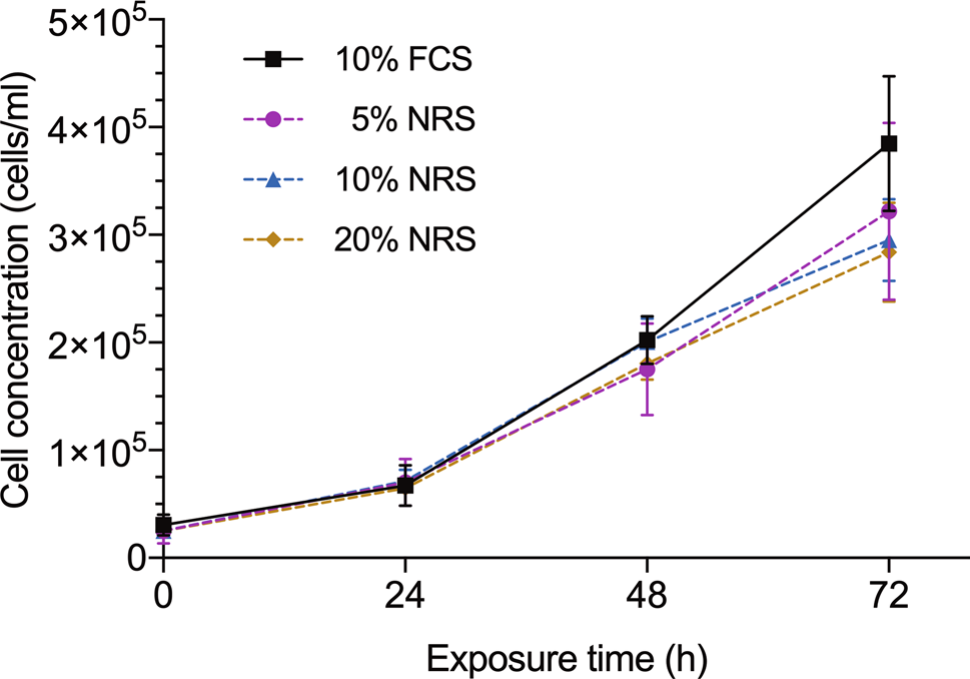
Proliferation curve of CC531 cell cultures. Cell concentration after 24, 48 and 72 h of exposure to different experimental conditions. Control (10% FCS) and normal rat serum (5%, 10%, 20%). Figure and analysis were performed using GraphPad Prism version 6.04 for Windows, GraphPad Software, San Diego, California USA, www.graphpad.com.

In order to select appropriate control conditions, first of all, we analysed the effect of adding serum obtained from normal rats (NRS) to CC531 cell cultures, comparing their growth rate at 72 h with that of cultures enriched with conventional 10% foetal calf serum (FCS). Though no differences were observed during the first 48 h (Fig. 1), looking at cell counts on the third day, none of the cultures enriched with NRS reached the cell numbers obtained with FCS, being the culture with the lowest concentration of NRS (5%) the one that was closest to the control.

Then, we compared the cultures enriched with HRS with the standard 10% FCS and 5% NRS. As it had happened with NRS, none of the cultures reached the values found in control cultures (FCS). And again, the proliferation rate closer to the control was seen with the lowest concentration of HRS (5%), while cultures enriched with higher concentrations (10% or 20%) of HRS showed significant smaller proliferative rates (Table 1).

**Table 1:** Cell count after 72 h of exposure to different concentrations of rat serum.

Serum concentration (v/v)	Normal rat serum (cell/ml)	Hepatectomized rat serum (cell/ml)	*p* value
20%	2.84 × 10^5^ ± 1.12 × 10^5^	1.27 × 10^5^ ± 0.32 × 10^5^	< 0.0001
10%	2.95 × 10^5^ ± 0.93 × 10^5^	2.25 × 10^5^ ± 0.40 × 10^5^	< 0.0001
5%	3.22 × 10^5^ ± 0.82 × 10^5^	3.20 × 10^5^ ± 0.70 × 10^5^	n.s
2.5%	4.32 × 10^5^ ± 0.75 × 10^5^	5.18 × 10^5^ ± 0.38 × 10^5^	< 0.0001
1%	3.98 × 10^5^ ± 0.72 × 10^5^	4.34 × 10^5^ ± 0.33 × 10^5^	0.0018

Cell count is expressed as media and standard deviation.

**Figure 2:**
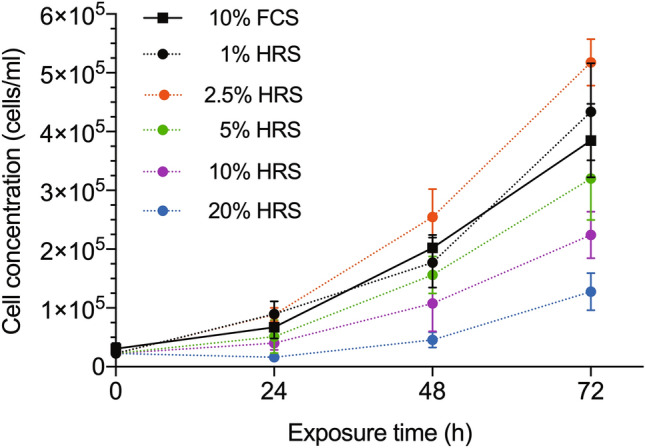
Proliferation curves of CC531 cell cultures enriched with serum from hepatectomized rats. Control (10% FCS) and hepatectomized rat serum (HRS; 1%, 2.5%, 5%, 10%, 20%). Figure and analysis were performed using GraphPad Prism version 6.04 for Windows, GraphPad Software, San Diego, California USA, www.graphpad.com.

**Figure 3:**
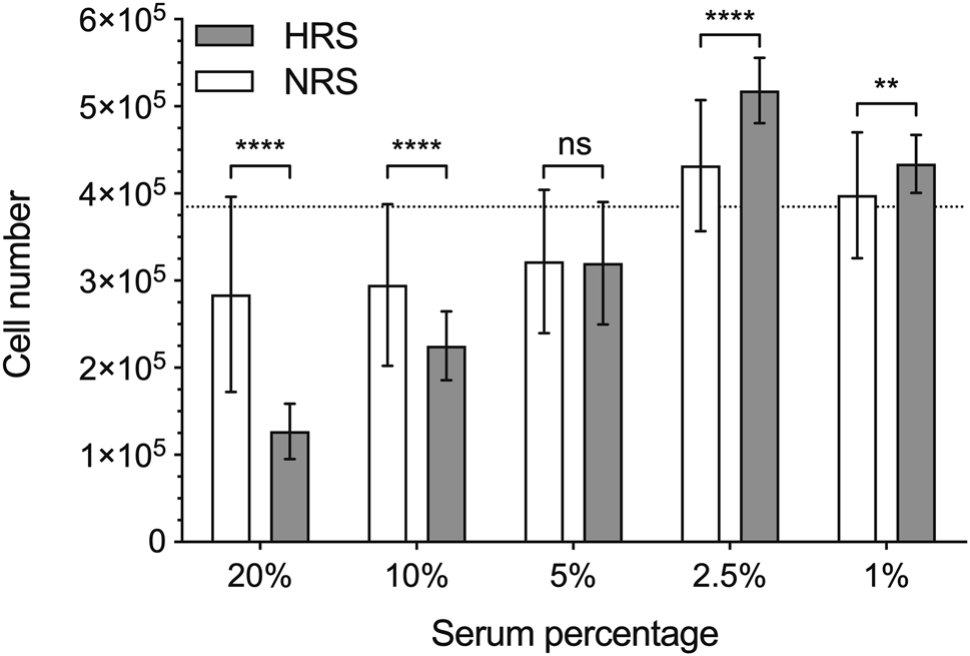
Proliferation curves of CC531 cell cultures enriched with low concentration of rat serum (normal rat serum, NRS: white; hepatectomized rat serum, HRS: grey). Dotted line represent cell numbers of cultures enriched with foetal calf serum (FCS). Figure and analysis were performed using GraphPad Prism version 6.04 for Windows, GraphPad Software, San Diego, California USA, www.graphpad.com.

Given these findings, we performed another set of experiments reducing the amount of rat serum added to the cultures (2.5%, 1%). With this lower concentrations, cultures receiving HRS achieved stronger proliferative rates than the control cultures (10% FCS), being 2.5% the concentration reaching the highest values (*p* < 0.0001) (Fig. 2). Moreover, at these low concentrations (1% and 2.5%), HRS also induced higher proliferative rates that their counterpart of NRS (*p* < 0.0001) which was not seen with greater concentrations (Fig. 3).

If we focus on the results obtained in cultures enriched with 2.5% rat serum, those cultures receiving HRS proliferated at quicker pace than those supplemented with NRS; the effect being statistically significant even at 48 h (*p* < 0.05).

### In vivo model assessment

Ultrasound examination of the animals on days 15th and 20th provided no useful information about tumour development, as it was not possible to accurately assess neither the size nor the number of tumour foci. When livers were removed on day 28th all of the animals had developed liver implants, regardless of whether they underwent PHx or not. Macroscopic examination of the liver showed that tumour foci were randomly distributed through the whole organ, without a specific pattern, though the concentration of tumour implants was lower in the paramedian lobe.

### Effect of 40% hepatectomy on tumour development

**Figure 4:**
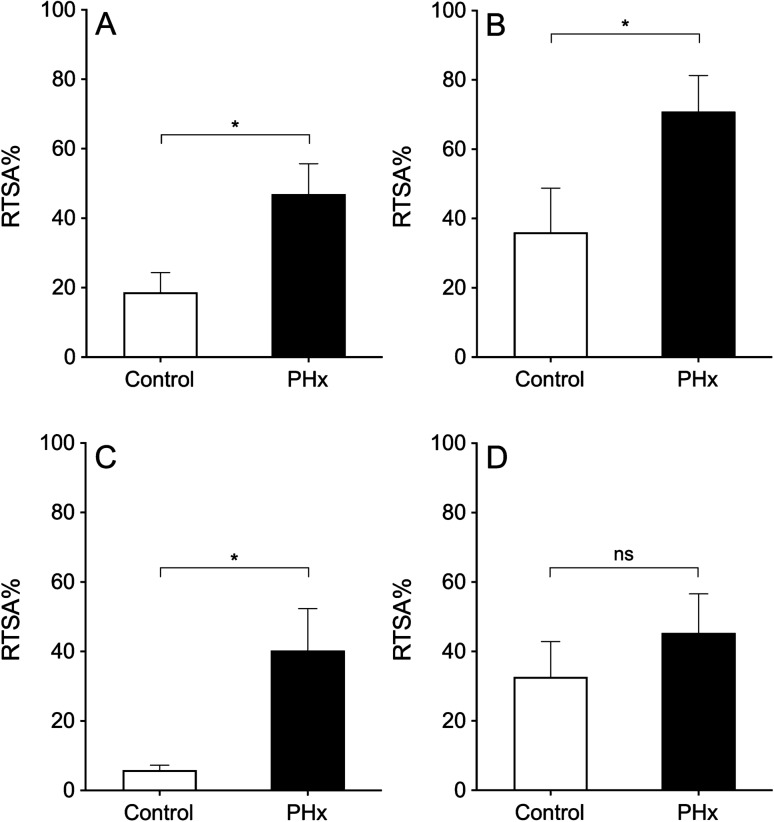
Relative tumour surface area percentage (RTSA%). Bar graphs of total liver mass RTSA% (**A**), caudate lobe RTSA% (**B**), paramedian lobe RTSA% (**C**) and right lateral lobe RTSA% (**D**) of both control (non-hepatectomized animal; white) and PHx (hepatectomized animal, black) groups. Asterisk show statistical differences (*p* < 0.05). Figure and analysis were performed using GraphPad Prism version 6.04 for Windows, GraphPad Software, San Diego, California USA, www.graphpad.com.

The percentage of liver area occupied by tumour tissue in the group of hepatectomized animals was 2.5 times higher than in the control group (47% vs. 18.7%, respectively, *p* < 0.05; Fig. 4A; Table 2). Analysing the liver lobes separately, in the paramedian and caudate lobes the relative tumour surface area percentage (RTSA%) was strongly increased in hepatectomized animals (40.3% vs. 5.9%; 70.9 vs. 36.0%, respectively). However, in the right lateral lobe, though the percentage was also slightly higher in the hepatectomized group (45.4% vs. 32.7%), this difference was not big enough to be statistically significant (*p* = 0.42; Fig. 4B–D).

**Table 2:** Relative tumour surface area occupied by CRCLM in total liver mass (TL-RTSA%), caudate lobe (CL-RTSA%), paramedian lobe (PL-RTSA%) and right lateral lobe (RLL-RTSA%).

	TL-RTSA%	CL- RTSA%	PL-RTSA%	RLL-RTSA%
Control group	18.73 ± 5.6%	36.02 ± 12.80%	5.9 ± 1.4%	32.69 ± 10.19%
PHx group	46.98 ± 8.8%	70.91 ± 10.35%	40.33 ± 12.1%	45.38 ± 11.24%
*p* value	0.015	0.04	0.03	0.42

## Discussion

The aim of this piece of work has been to validate an animal model in which hepatectomy clearly enhance the growth of tumour foci in the liver, and simultaneously assess the effect of serum obtained from hepatectomized animals on the proliferation rate of the same cell line. This model would be a very handy tool to test new molecules with the capability of slowing tumour growth while not hindering liver regeneration. Preliminary tests could be carried out in vitro, and those proving to be effective could be immediately be evaluated in the in vivo model.

It could be argued that the ideal animal model to study this phenomenon would be based on naturally-appearing primary CRC which subsequently develops CRCLM. Then, PHx could be performed to remove metastases, and the recurrence of liver metastases could be studied. However, such a model based on natural or genetic predisposition to develop primary CRC and then CRCLM has several drawbacks. Major problems that discourage the use of such a model include: low rates of development both of primary tumours and metastases, long time needed for metastatic disease to become evident, a limited number of models (syngeneic and xenograft), metastatic disease not being confined to a single location and the asynchronous development of metastatic disease, as well as greater associated costs of animal housing^22–24^.

Chemical induction has also been accepted as an appropriate tool for tumour induction. Various compounds, including dimethylhydrazine and its metabolites methylnitronitrosoguanidine, N-Nitroso-N-methylurea and azoxymethane, are able to induce tumour and/or metastases growth in laboratory animals^25–29^. These compounds have alkylating activity, which cause breakage of DNA chains, abnormal pairing of bases, and inhibition of cell division, finally, resulting in cell death. Despite these products being well known, they have several disadvantages when used to induce tumours. For example, long induction time, low rates of tumour development and undefined site of tumour development, meaning that larger numbers of animals would be required for each experimental group. Furthermore, as these chemical products are carcinogenic in humans, the risk of researcher exposure should be taken into account.

Heterotopic implantation of cancer cells is also a validated model to induce tumour development in laboratory animals, particularly in rats and mice. Cells may be implanted directly into the liver parenchyma, via subcapsular injection, and this approach achieves a high tumour development rate (up to 60–70% of the injected animals)^30–32^. But this model is not appropriate for our purposes because it only allows the development of single well-defined and localized tumour implants, and also it lacks the process of tumour cell dissemination through the vascular tree and subsequent extravasation to produce metastatic disease in the liver^33^. Another heterotopic-implantation method to induce CRCLM development is intrasplenic injection. Published data regarding the success rate with this model is, however, very heterogeneous, ranging between 20 and 100%^34,35^.

For this piece of work, we chose intrasplenic injection because it mimics, as closely as may be possible, the natural haematogenous dissemination pathway through the portal vein of CRC cells from primary colorectal tumours, as well as all the other natural events involved in the spreading of metastases, such as extravasation, implantation, extracellular matrix remodelling and CRCLM growth and development.

The immune barrier is another factor to be considered when performing tumour induction by cell injection. To achieve a successful model, allo- or xeno-rejection has to be avoided. This problem can be overcome by the use of cell lines that grow in syngeneic hosts, also called isotransplantation^36^, or using immunocompromised host, animals which have an immune system that is suppressed or depleted and does not respond when cells or tissues from other species are implanted^36,37^.This last approach deprives the animal of the immune system, which is known to be the main organizer of regenerating processes and plays a very important role in cancer.

The last important aspect concerning the model we have used is the clamping of the portal pedicle of the left lateral lobe (LLL). Although clamping the LLL may elicit the production of stimulating cytokines that could interfere with the procedure for tumour induction, the period of time that the LLL remains clamped is too short (5 min) to have a substantial consequence, considering the large regenerative stimulus induced by hepatectomy^38,39^.

As the immune system plays an important role in cancer, we decided to avoid interfering with it. For this reason, we chose a CRC cell line (CC531) which is syngeneic to WAG/RijHsd rats. When CC531 cells are injected into the spleen of this strain of rats, rejection seldom occurs^40^ and a high rate of success is achieved. In fact, in this study, we saw no cases of rejection at all, and a 100% success rate in CRCLM development. The fact that tumour foci were observed in every liver lobe is in consonance with the natural pattern of metastasis development, which reinforces our idea of it being a good experimental model for studies on liver metastases. Nonetheless, it is also true that this model lacks the genetic variability which is observed in human tumours^41^. Moreover, Robertson et al. sustain that as rat liver architecture and homeostasis are quite different from those in humans, caution should be exercised with any extrapolation of results from this model to a clinical setting^37^.

Following liver resection, a vigorous peak of HGF is found in serum, which then decreases to a plateau which lasts all the period of hepatocyte proliferation; the beginning of this plateau matches the first peak of DNA synthesis^42^. To obtain rat serum rich in HGF we chose a 40% hepatectomy, because we plan to mimic liver surgery for metastases removing the LLL. Following a 40% hepatectomy in the rat, the first peak in DNA synthesis is reached after 40 h^20,21,43–45^, so that was the timing selected to retrieve the serum from the animals.

Our finding that there is a certain concentration of serum in the culture which elucidates the higher proliferative rate of cancer cells, while values above or below show a lesser effect is in accordance with what has been published by other authors^45,46^. Liu et al. exploring the effect of different concentrations of serum on the growth of mouse fibroblasts in culture, found that the highest proliferation was obtained with 20%, with higher or lower concentrations growth was much lesser^47^.

The fact that the optimum serum concentration for the stimulation of tumour cell proliferation is lower when using serum from hepatectomized animals suggests that there is something in it which make it more a powerful stimulus for cell growth. It is well known that following any kind of surgery there is an increase in GF levels in serum, both in humans^48,49^ and in rats^49,50^. HGF increases following lung surgery^51^, but the increment is quite greater following liver resection (from 50 to 250 ng/ml)^42^. To avoid inappropriate cell duplication when GF are produced in our organism, mammalian cells need an activator to respond to GF^50^, but cancer cells lack this protective mechanism. These facts support the hypothesis that GF produced during surgery for CRCLM could be responsible of the high frequency of recurrence reported^46,52^.

In fact, the experiments we have carried out in vivo have shown that 40% PHx strongly increases CRCLM development. Authors including Panis et al., García-Alonso et al., Krause et al. and Harun et al. have obtained similar results. Specifically, in an intraportal induction model of CRCLM (DHDK12 CRC cell line and syngeneic BDIX rats), Panis et al. demonstrated that the number of rats developing liver metastases tripled when the rats underwent 70% PHx (62% vs. 20%, *p* < 0.05)^53^. Working with S4MH (a rhabdomyosarcoma cell line, syngeneic for WAG rats) and performing 40% PHx, we reported stimulatory effects on liver metastasis development, compared to that observed in controls (non-hepatectomised)^54^. Regarding the behaviour of each liver lobe, our results are quite similar to this latter study. The percentage of the liver parenchyma occupied by tumour implants was significantly higher in the paramedian and caudate lobes in hepatectomised animals (33% vs. 24% and 47% vs. 17%, respectively); it was also slightly higher in the right lateral lobe, though the difference did not reach significance (20% vs. 17%). Krause et al., using CC531 cells and WAG rats, reported an 2.8-fold increase in tumour volume in their PHx group, estimated by MRI^55^.

As in any other research carried out in animals, it remains to be proved that our results can be translated into clinical practice. There has been some recent clinical research focused on the effects of liver resection on tumour growth and recurrence of liver metastases. Margonis et al. have reported that those patients with a higher kinetic growth rate assessed in the late regeneration phase are associated with increased risk of intrahepatic recurrence. On the other hand, Hamm et al. in a retrospective study have found that patients with lower liver regeneration showed earlier recurrence of liver metastases; but they also reported that the extent of liver regeneration after major hepatectomy did not modify overall survival of their patients. It is our believe that beyond this apparent paradox neither of the studies deny there is an effect of liver resection and surgery in general on cancer progression. If a molecule able to block the protumour effect of hepatectomy in rats were found, then the important translational question should have to be arisen.

In summary, the results obtained from this experimental model and our preliminary results are in accordance with data published by other authors, demonstrating that liver resection to remove the macroscopic metastases, though effective in improving short- and medium-term survival, carries the risk of promoting the proliferation of any tumour cells remaining in the patient. Once we have demonstrated that there is a parallelism between our in vivo and in vitro models, it can be used to test novel agents which could hinder the proliferative effect of liver resection on tumour growth while not affecting liver restoration following this surgery.

## Conclusions

In our model, hepatic resection performed on tumour-bearing livers promotes both the growth of cancer cells in the remnant liver parenchyma and the proliferation of colorectal cancer cells in vitro. This finding supports the hypothesis that molecules released to the plasma after partial liver resection are responsible for cancer recurrence following surgical oncological treatments, and offers a useful experimental model to check new treatments.

## Material and methods

All procedures involving animals were performed in strict accordance with the ARRIVE guidelines and followed the recommendations of current national legislation on experiments involving animals or biological agents. All protocols were approved by the Ethics Committee on Animal Experimentation (CEEA) (ref. number: M20/2015/054) and Ethics Committee for Research involving Biological Agents and Genetically Modified Organisms (CEIAB) (ref. number: M30/2018/022) of the University of the Basque Country (UPV/EHU).

### Animals

The experiment was carried out in 24 three-month-old male WAG/RijHsd rats, weighing 270–300 g. Animals were kept in a temperature- and humidity-controlled room with a 12-h light/dark cycle and free access to standard laboratory diet and water. Animals were randomly allocated to one of three groups: (1) 40% hepatectomy (to obtain serum for in vitro experiments; n = 6), (2) midline laparotomy and tumour inoculation (sham-operated group, n = 9), (3) midline laparotomy and tumour inoculation & PHx (PHx group, n = 9). All the surgical procedures were carried out under anaesthesia with isoflurane 1.5%.

The animals in the first group underwent 40% PHx, removing the LLL through a midline laparotomy. Forty hours later, they were anesthetized, had blood collected from the abdominal cava vein, and were then sacrificed. After centrifugation for 10 min at 3000 rpm, the serum was obtained and stored at − 20 °C until use.

All the other animals received an intrasplenic injection of 0.5 ml of 500,000 CC531 cells/ml cell suspension to induce liver cancer implants. A midline incision was performed to expose both the liver and the spleen. The LLL vascular pedicle was blocked with a Yasargil vascular microclamp, to prevent cancer cells entering this lobe (as such cells would be lost in animals undergoing 40% PHx). Then, the spleen was exposed and the cells were slowly inoculated into the organ. Once the injection had been completed, both the liver and the spleen were returned to the abdominal cavity and the laparotomy was closed with a running suture. Five minutes later, the laparotomy was reopened to excise the spleen and remove the microclamps. At this point, the LLL was exposed; in half of the animals, it was returned to the abdomen, while in the other half it was removed. Then, the abdominal wall was closed with interrupted stiches, and meloxicam (2 mg/kg) was administered. The animals were kept under observation until full recovery. On days 15 and 20, an ultrasound examination of the liver was performed, looking for any tumour foci. These examinations were carried out using a MyLab 60 Xvision system (Esaote, Genoa, Liguria, Italy), equipped with a multifrequency linear probe operated at 18 MHz and the focus was set at a depth of between 0.5 and 1.5 cm. On day 28, under isoflurane anaesthesia, the liver was removed and fixed in 10% formaldehyde, and the animals sacrificed.

### Relative tumour surface area

**Figure 5:**
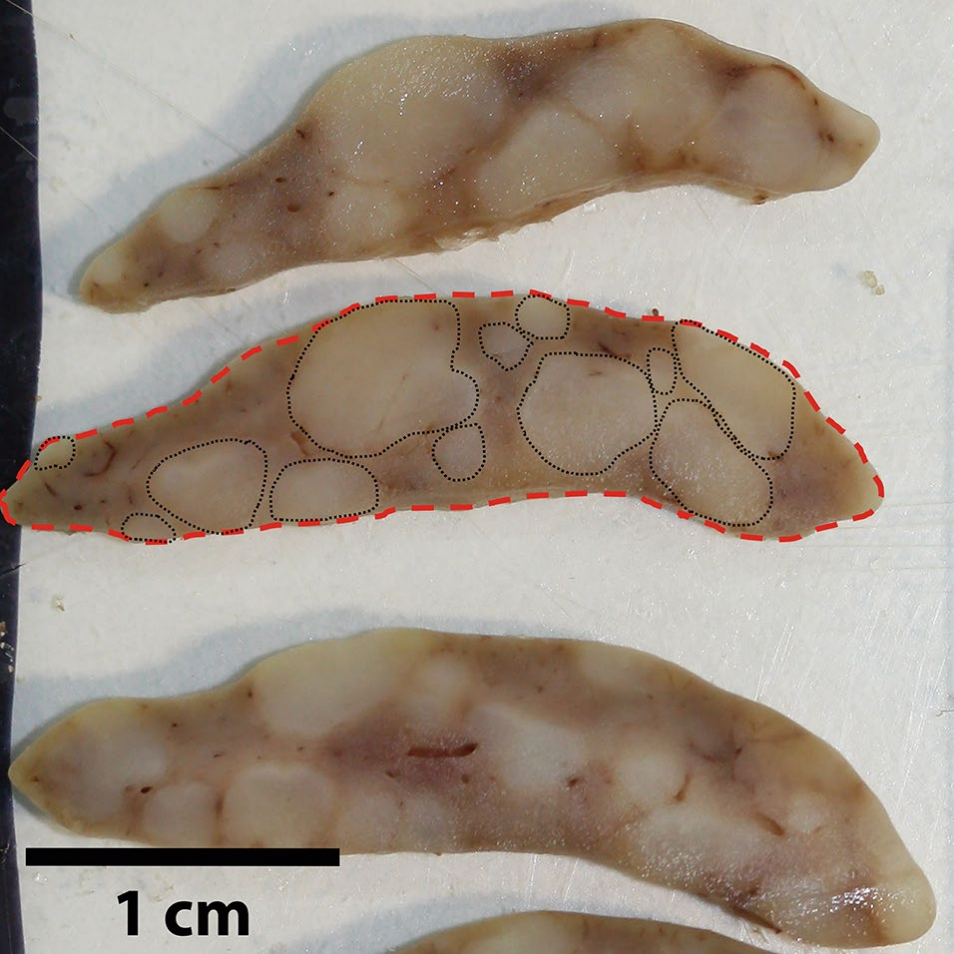
Measurement of absolute tumour surface area (ATSA) (encircled by discontinuous black lines) and absolute liver surface area (ALSA) (shaded green and encircled by a discontinuous red line) in each slice using ImageJ, version 1.52p for windows, U.S. National Institutes of Health, Bethesda, Maryland.

Each liver lobe was identified, excised and sliced into 1 mm slices. Then, all slices from each lobe were photographed separately and labelled with a blinded code. Using ImageJ, (version 1.52p for windows, U.S. National Institutes of Health, Bethesda, Maryland USA), absolute tumour surface area (ATSA) and absolute liver surface area (ALSA) were measured in square pixels (p^2^), as shown in Fig. 5. Then, RTSA% was calculated [RTSA% = (ATSA/ALSA) × 100]. Once the measurements had been completed, the blind was broken and the data sorted by individual animal and experimental groups.

### In vitro assessment of hepatectomized rat serum

To assess the potential of HRS to enhance cell proliferation, a series of in vitro cell proliferation studies were carried out. CC531 cells were incubated at 37 ºC and 5% CO_2_ in RPMI-based culture medium supplemented with 10% (v/v) foetal calf serum (FCS), antibiotics (100 IU/ml penicillin and 100 µg/ml streptomycin) and antimycotics (0.25 µg/ml amphotericin B). Once the culture reached semi-confluence, the cells were harvested and seeded on 24-multiwell culture plates at 30,000 cells/ml. The control wells were supplemented with 10% FCS, while those of experimental groups were supplemented with either 5, 10 or 20% NRS (obtained from non-hepatectomized rats) or 1, 2.5, 5, 10, or 20% HRS. Cell proliferation was assessed using NucleoCounter NC-100 (Chemometec, Allerod, Denmark) and xCELLIgence RTCA SP (ACEA Biosciences Inc. San Diego, California, USA).

### Statistical analysis

The normality of the data was checked with the Kolmogorov–Smirnov test, and results were summarized using means and standard deviations. Comparisons between two groups were carried out using two-tailed t-tests with a confidence level of 95% (for in vivo studies). When three or more groups were compared (for in vitro studies), an analysis of variance (ANOVA) was performed, and Newman–Keuls multiple comparison test for between-group comparison was performed when ANOVA showed significant differences (*p* < 0.05). Statistical analyses were performed using GraphPad Prism version 6.04 for Windows, GraphPad Software, San Diego, California USA.

### Ethics approval and consent to participate

This project has been approved by the Ethics Committee on Animal Experimentation (CEEA) (ref. number: M20/2015/054) and Ethics Committee for Research involving Biological Agents and Genetically Modified Organisms (CEIAB) (ref. number: M30/2018/022) of the University of the Basque Country (UPV/EHU).
